# Scintillation properties of Ca co-doped L(Y)SO:Ce between 193 K and 373 K for TOF-PET/MRI

**DOI:** 10.1186/2197-7364-1-S1-A10

**Published:** 2014-07-29

**Authors:** David N ter Weele, Dennis R Schaart, Pieter Dorenbos

**Affiliations:** Faculty of Applied Sciences, Delft University of Technology, Mekelweg 15, Delft, 2629 JB the Netherlands

Time-of-flight Positron Emission Tomography (TOF-PET) and TOF-PET/MRI require scintillators with high light yield, short decay time, and short rise time in order to obtain high timing resolution. LSO:Ce and LYSO:Ce are commonly used. Ca co-doped LSO:Ce shows improved scintillation properties. The decay time constant of LSO:Ce,0.2%Ca (~33 ns) is shorter than standard LSO:Ce (~38-40 ns), and it has about 15% higher light yield. We measured scintillation pulse shapes and photoelectron yields of LSO:Ce, LSO:Ce,0.2%Ca, LYSO:Ce, LYSO:Ce,20ppmCa, LYSO:0.11%Ce,0.2%Mg, and LYSO:0.2%Ce,0.2%Ca at temperatures ranging from 193 K to 373 K. To study rise times we built a set-up in which samples are excited by 100 ps (FWHM) x-ray pulses.

**Figure 1:**
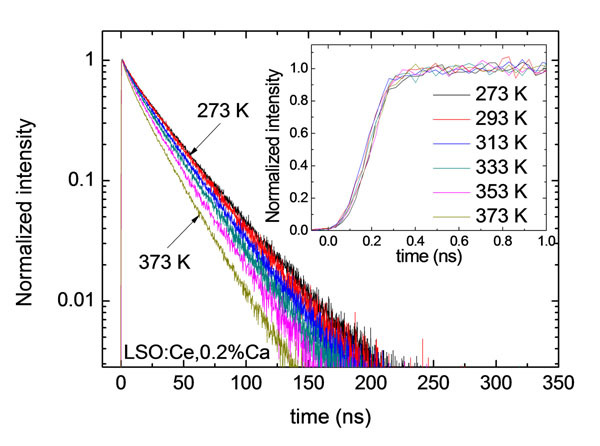
Scintillation pulse shape of LSO:Ce,0.2%Ca.

**Figure 2:**
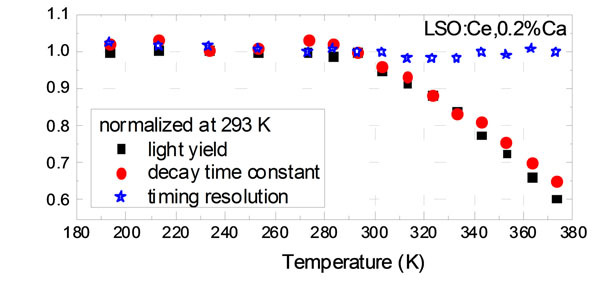
Normalized scintillation properties as a function of temperature.

Figure 1 shows that the scintillation rise time of LSO:Ce,0.2%Ca is constant between 273 K and 373 K, while the decay time constant decreases. Figure 2 shows that the light yield of LSO:Ce,0.2%Ca decreases with rising temperature. We noticed that the decrease in light yield is entirely caused by non-radiative decay of excited Ce^3+^ centres. This quenching mechanism does not worsen the timing resolution, as opposed to recombination of electron-hole pairs. It follows that the timing resolution of LSO:Ce(,0.2%Ca) is constant over the entire temperature range, in accordance with the timing model described in [1].
